# Host Lipid Mediators in Leprosy: The Hypothesized Contributions to Pathogenesis

**DOI:** 10.3389/fimmu.2018.00134

**Published:** 2018-02-02

**Authors:** Carlos A. M. Silva, John T. Belisle

**Affiliations:** ^1^Mycobacteria Research Laboratories, Department of Microbiology, Immunology, and Pathology, Colorado State University, Fort Collins, CO, United States

**Keywords:** leprosy, *M. leprae*, resolvin, leukotriene, lipoxin, prostaglandin, immune responses, clinical spectrum

## Abstract

The spectrum of clinical forms observed in leprosy and its pathogenesis are dictated by the host’s immune response against *Mycobacterium leprae*, the etiological agent of leprosy. Previous results, based on metabolomics studies, demonstrated a strong relationship between clinical manifestations of leprosy and alterations in the metabolism of ω3 and ω6 polyunsaturated fatty acids (PUFAs), and the diverse set of lipid mediators derived from PUFAs. PUFA-derived lipid mediators provide multiple functions during acute inflammation, and some lipid mediators are able to induce both pro- and anti-inflammatory responses as determined by the cell surface receptors being expressed, as well as the cell type expressing the receptors. However, little is known about how these compounds influence cellular immune activities during chronic granulomatous infectious diseases, such as leprosy. Current evidence suggests that specialized pro-resolving lipid mediators (SPMs) are involved in the down-modulation of the innate and adaptive immune response against *M. leprae* and that alteration in the homeostasis of pro-inflammatory lipid mediators versus SPMs is associated with dramatic shifts in the pathogenesis of leprosy. In this review, we discuss the possible consequences and present new hypotheses for the involvement of ω3 and ω6 PUFA metabolism in the pathogenesis of leprosy. A specific emphasis is placed on developing models of lipid mediator interactions with the innate and adaptive immune responses and the influence of these interactions on the outcome of leprosy.

## Introduction

Leprosy is a chronic granulomatous disease driven by interactions of the human host with *Mycobacterium leprae* an obligate intracellular pathogen that infects macrophages and Schwann cells of the peripheral nervous system. *M. leprae* is the only mycobacterial infection that causes widespread demyelinating neuropathy, which results in severe and irreversible nerve tissue damage. The prevalence of leprosy is gradually decreasing in many countries due to multidrug therapy (MDT) (1). However, the rates of new case detection remain relatively stable in developing countries (1). India and Brazil are the countries that exhibit the highest incidence and account for 60 and 13% of the global new cases of leprosy, respectively (1).

Leprosy is well known for its bi-polarization of the immune response, and it is established that the nature and magnitude of the host immune response against *M. leprae* are critical factors for the pathogenesis of leprosy and its varied clinical manifestations. At one end of the spectrum, tuberculoid (TT) disease is typified by strong T-helper type 1 (Th1) cellular immunity and low bacterial load (2–4). This response promotes the protection against the pathogen via interferon-gamma (IFN-γ) activation of macrophage anti-microbicidal mechanisms (5). These patients also present robust T-helper type 17 (Th17) activity (6) that stimulates macrophages and enhances Th1 responses (7). The other end of the spectrum, lepromatous leprosy (LL), is characterized by a low or even absent Th1 response (8) but robust T-helper type 2 (Th2) and humoral responses. The diminished Th1 response in LL is partially explained by the highly suppressive activity of T regulatory (Treg) cells and the reduced frequency of Th17 cells (4, 6). Consequently, these patients manifest the most severe form of the disease and are unable to control *M. leprae* growth (2). Between these two clinical forms, patients with intermediate immune responses develop borderline clinical forms: borderline tuberculoid (BT), borderline-borderline (BB), and borderline lepromatous (BL). BT patients present with a dominant IFN-γ response, and also a higher activity of Th17 cells (6), while BL patients exhibit T-cell anergy, because of the higher frequency of Treg cells (4, 6), and a higher production of interleukin-4 (IL-4) (9–11). Peripheral neuropathy can occur in all clinical forms of leprosy but is most pronounced in patients who present with an exacerbated acute immune-inflammatory response, designated type 1 reaction (T1R). Multiple studies indicate that pathogenic CD8^+^ and CD4^+^ T cell responses (12–14) and production of nitric oxide (NO) in *M. leprae*-infected macrophages are related with nerve injury in leprosy patients (15). Thus, the human immune response against *M. leprae* is involved with key aspects of leprosy pathogenesis.

Metabolomic-based studies reveal that *M. leprae* infection promotes several modifications in human metabolism. The most prominent of these metabolic changes is a correlation between the spectrum of clinical forms of leprosy and the metabolism of ω3 and ω6 polyunsaturated fatty acids (PUFAs) (16–18). Of particular interest are the PUFA-lipid mediators: prostaglandin E_2_ (PGE_2_), prostaglandin D_2_ (PGD_2_), leukotriene B_4_ (LTB_4_), lipoxin A_4_ (LXA_4_), and resolvin D1 (RvD1). Both PGE_2_ and PGD_2_ are found in elevated levels in the sera of LL patients as compared to BT patients (17). Additionally, PGD_2_ levels are increased in leprosy patients with T1R, while PGE_2_ levels decrease in patients with a T1R (18). BT and LL patients have similar levels of the pro-resolving lipid mediators, LXA_4_ and RvD1 (17). However, when compared with healthy individuals, the levels of LXA_4_ and RvD1 are elevated in the sera of BT and LL patients. In patients with T1R, the level of RvD1 is significantly decreased, as is the ratio of LXA_4_/LTB_4_ (18).

It is well established that lipid mediators derived from the metabolism of ω3 and ω6 PUFAs are able to modulate the innate and adaptive immune responses (19–26). Thus, we posit that the PUFA-derived lipid mediators are important factors in the pathogenesis of leprosy. The objectives of this review are to bring together metabolic and immunological data that support our hypothesis and to provide an understanding of how lipid mediators potentially function across the spectrum of disease. Specifically, we will focus the review on the five lipid mediators (PGE_2_, PGD_2_, LTB_4_, LXA_4_, and RvD1) found to be differentially produced in leprosy patients (17, 18).

## A Brief Review of the Relevant Lipid Mediators

**Figure 1:**
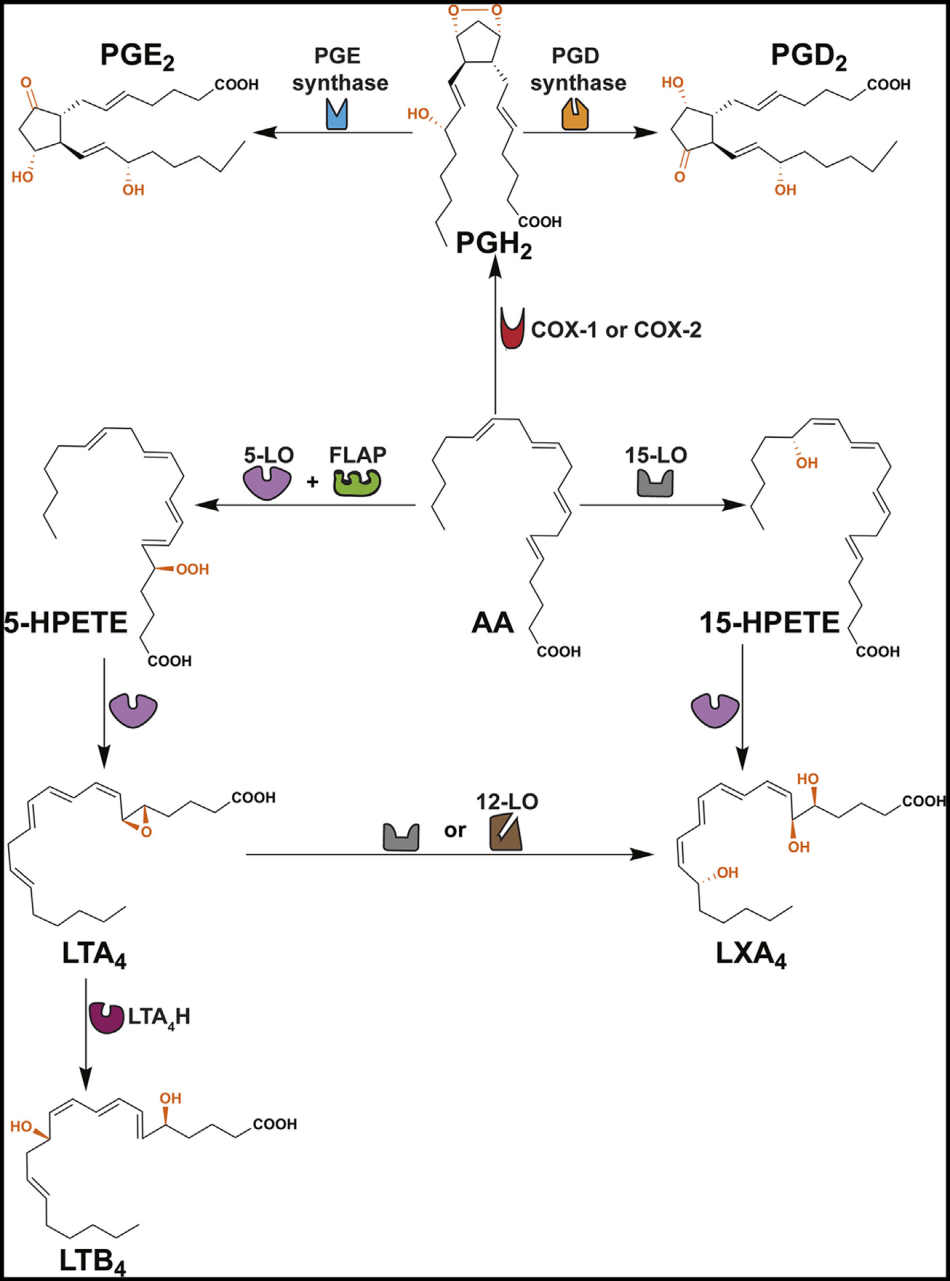
Formation of PGD_2_, PGE_2_, LTB_4_ and LXA_4_. This scheme shows that arachidonic acid (AA) is converted to several ω6 PUFA-derived lipid mediators through cyclooxygenase (COX) and lipoxygenase (LO) pathways. COX enzymes (constitutive COX-1 or inducible COX-2) exhibit a COX activity that incorporates two molecules of oxygen into AA to form PGG_2_ (not shown) and peroxidase activity that catalyzes a 2-electron reduction of PGG_2_ to PGH_2_. PGH_2_ is the direct precursor of PGD_2_ and PGE_2_. Formation of LTB_4_ occurs via the precursors 5-HPETE and LTA_4_. LXA_4_ is derived from 15-HPETE and/or LTA_4_. FLAP, 5-lipoxygenase-activating protein; LTA_4_H, leukotriene A_4_ hydrolase.

The ω6 PUFA, arachidonic acid (AA), is the precursor for a variety of lipid mediators (prostaglandins, leukotrienes, lipoxins, and thromboxanes) that exhibit immune-inflammatory functions (Figure 1; Table 1) (26–28). Importantly, AA can be metabolized by three separate pathways: cyclooxygenase (COX) pathway, lipoxygenase (LO) pathway, and epoxygenase pathway (the latter is not discussed in this review) (Figure 1) (29).

**Table 1:** Functions of the lipid mediators discussed in this review.

Lipid mediators	Receptor(s) and cell expression	Functions
Leukotriene B_4_	BLT1 – neutrophils, monocytes/macrophages, dendritic cells, mast cells, effector CD8^+^ T cells, naive CD4^+^ T cells, differentiated T-helper type 1 (Th1), T-helper type 2 (Th2), and T-helper type 17 (Th17) cells, and endothelial cells (30, 31) BLT2 – expressed ubiquitously (30, 31)	Recruit neutrophils, monocytes and macrophages (30) Enhance Th1 response (22) Recruits Th1, Th2, and Th17 cells (32, 33) Enhances TNF-α expression and also the production of pro-inflammatory cytokines associated with Th1 responses [interferon-gamma (IFN-γ) and interleukin (IL)-12] (21, 22)
Prostaglandin E_2_	EP1 – endothelial cells (34) EP2 – mast cells, neutrophil, naive T cells, monocytes, macrophages, Th17 cells, and endothelial cells (34–37) EP3 – platelets, mast cells, monocytes, and endothelial cells (34, 37) EP4 – mast cells, eosinophils, monocytes, dendritic cells, naive T cells, Th1 cells, Th17 cells, B lymphocytes, and endothelial cells (35–37)	Promotes local vasodilation, attraction and activation of neutrophils, macrophages, and mast cells at early stages of inflammation (38) Regulates the production of IL-23 in dendritic cells (23) Inhibits the synthesis of IL-12 in dendritic cells (19) Impairs the proliferation of T cells (39, 40) Regulates the production of nitric oxide (41) Modulates Th1 cells differentiation (24, 42–44) Promotes the expansion of T regulatory (Treg) cells (45) Up-regulates the transcription factor FOXP3 (46) Inhibits the activation of macrophages by IFN-γ (47) Induces apoptosis (48, 49)
Prostaglandin D_2_	DP1 – mast cells, monocytes, and immature and mature dendritic cells (19, 50) CRTH2 – Th2 cells, basophils, eosinophils, mast cells, macrophages, and dendritic cells (19, 51–54)	Promotes the myelination of neurons (55) Induces vasodilation, erythema, edema and induration (56–58) Down-modulates the synthesis of IL-12 in dendritic cells (19, 59) Enhance the ability of Th2 cells to produce IL-2, IL-4, IL-5, and IL-13Reduces the numbers of CD4^+^ and CD8^+^ T cells that produces IL-2 and IFN-γ (60, 61) Induces chemotaxis of Th2 cells, eosinophils, and basophils (62)
Lipoxin A_4_	ALX/FPR2 and GPR32 – monocytes macrophages, neutrophils, and T cells (Th1, Th17, and Tregs) (26, 63)	Inhibits the recruitment of neutrophils (64) Promotes macrophage efferocytosis (65) Down-regulates Th1-derived cytokines like IFN-γ, TNF-α, and IL-6 (20, 21, 66, 67) Induces the synthesis of the anti-inflammatory cytokine IL-10 (66) Inhibits the synthesis of LTB_4_ (68)
Resolvin D1	ALX/FPR2 and GPR32 (see Lipoxin A4) (26, 63)	Shortens resolution of inflammationInhibits the recruitment of leukocytes (28, 69) Down-modulates the production of TNF-α, IL-6, IL-8, IFN-γ, and IL-12 (70–72) Up-modulates the production of IL-10 (70) Efferocytosis (73) Inhibits LTB_4_ production (68) Decreases the capacity of Th1 and Th17 cells to produce IFN-γ and IL-17, respectively; prevents Th1 and Th17 generation from naive CD4 T cells; promotes the *de novo* generation of Treg cells; and induces the expression of CTLA-4 (26)

The COX pathway converts AA into prostaglandins via two isoforms of COX, COX-1 and COX-2 (Figure 1) (29). Both enzymes convert AA into PGG_2_, which is reduced to PGH_2_ and then converted to PGD_2_ or PGE_2_ by PGD or PGE synthase, respectively (Figure 1) (74). PGE_2_ and PGD_2_ are involved with the early stages of inflammation, and it is well established that both lipid mediators exhibit a dual role in immune-inflammation due to their capacities to exert pro- and anti-inflammatory responses (Table 1) (38, 75). This might be partially explained by the fact that both prostaglandins are recognized by more than one prostaglandin receptor (PGE_2_ – EP1, EP2, EP3, and EP4; PGD_2_ – DP1 and CRTH2) (see Table 1) (19, 37, 51, 52). Moreover, PGD_2_ and its metabolites (e.g., 15d-PGJ_2_) are ligands for the peroxisome proliferator-activated receptor gamma (PPAR-γ) (76, 77).

The LO pathway converts AA to leukotrienes and lipoxins (29). The production of LXA_4_ and LTB_4_ is dependent on 5-LO that converts AA to leukotriene A_4_ (LTA_4_) via 5-hydroperoxyeicosatetraenoic acid (5-HPETE) (Figure 1) (78–82). Subsequently, LTA_4_ hydrolase (LTA_4_H) catalyzes the conversion of LTA_4_ to LTB_4_ (83) and platelet-derived 12-LO or 15-LO uses LTA_4_ as a substrate for the production of LXA_4_ (Figure 1) (84, 85). LTB_4_ is involved in the initiating steps of the immune-inflammatory response and exerts its pro-inflammatory functions through two G-protein-coupled receptors BLT1 and BLT2 (Table 1) (86). More specifically, LTB_4_ has the capacity to act as a chemoattractant for leukocytes, activate inflammatory cells (30), and favor Th1 and Th17 responses (Table 1) (21, 32, 33, 87–89). In contrast, LXA_4_ is a specialized pro-resolving lipid mediator (SPM) that acts via the G-protein-coupled receptors ALX/FPR2 and GPR32 (Table 1) (63). An imbalance between the levels of LXA_4_ and LTB_4_ exacerbate the immune-inflammatory response and/or favor pathogen survival, including mycobacterial infections (21, 90). Importantly, the SPMs promote the resolution phase of inflammation by impairing the recruitment of leukocytes, stimulating the engulfment of apoptotic cells by phagocytes (known as efferocytosis) and inducing tissue repair (28, 69).

**Figure 2:**
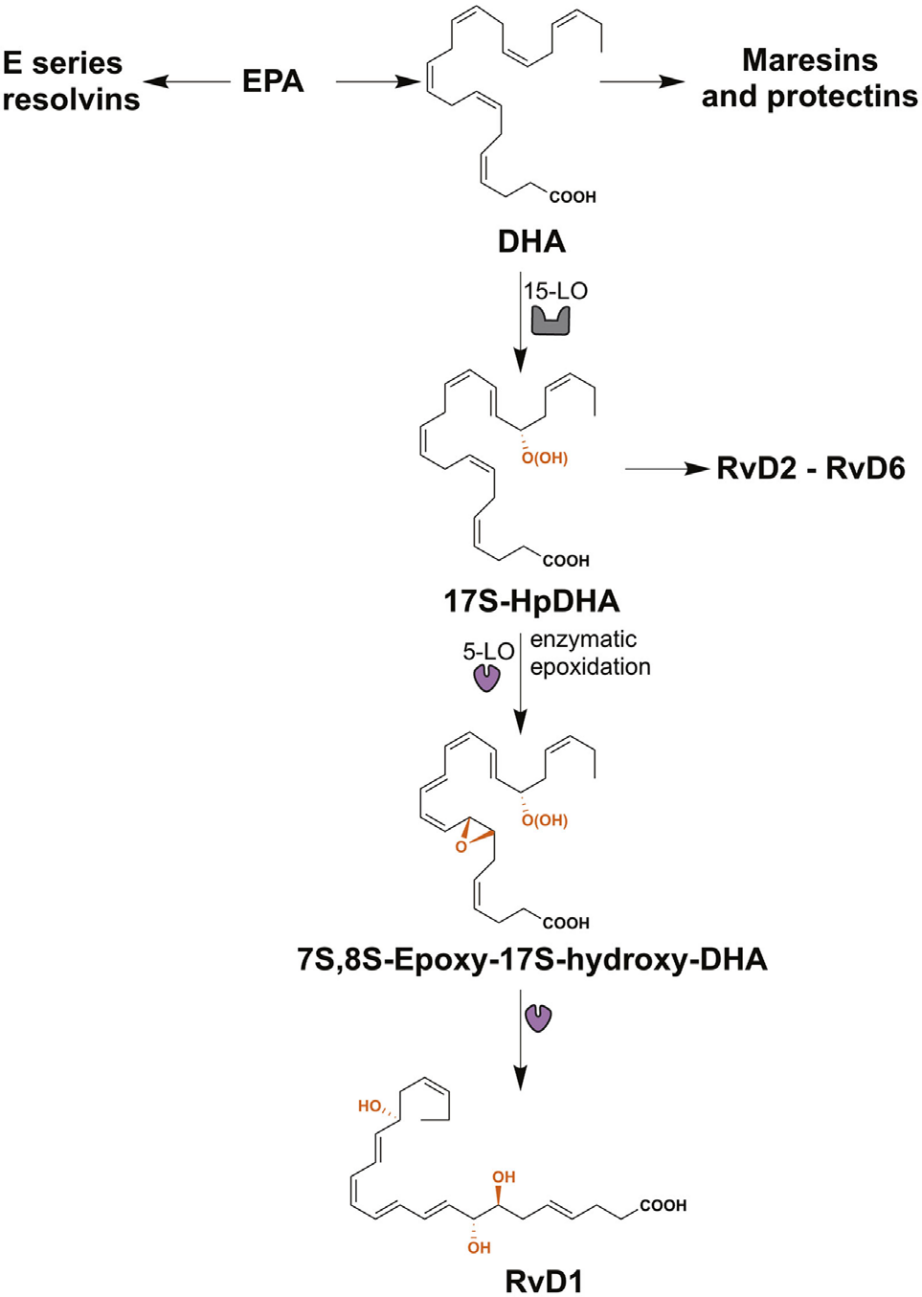
The biosynthesis of resolvin D1 (RvD1). The resolvins from the E-series (resolvins E1–E3) are synthesized from eicosapentaenoic acid (EPA), while maresins (maresin-1 and maresin-2), protectins (protectin-1 and neuroprotectin-1), and resolvins of the series-D (resolvins D1–D6) are produced from docosahexaenoic acid (DHA). RvD1 is generated from the sequential oxygenation of DHA, a process catalyzed by 15-lipoxygenase (15-LO) and 5-lipoxygenase (5-LO). The initial conversion of DHA to 17S-HpDHA is catalyzed by 15-LO, followed a second lipoxygenation via 5-LO, which gives a peroxide intermediate that is transformed to 7*S*-,8*S*-epoxid-17*S*-hydroxy-DHA. Subsequently, the enzymatic hydrolysis of this compound generates the trihydroxylated product RvD1.

Lipid mediators derived from the essential ω3 PUFAs, eicosapentaenoic acid (EPA), and docosahexaenoic acid (DHA) include the resolvins, maresins, and protectins, all of which are SPMs (Figure 2) (28). The E-series resolvins (resolvins E1 to E3) are synthesized directly from EPA, while maresins (maresin-1 and maresin-2), protectins (protectin-1 and neuroprotectin-1), and D-series resolvins (resolvins D1 to D6) are produced from DHA (Figure 2). However, DHA itself can be produced from EPA by two elongation steps, desaturation and subsequent β-oxidation in the peroxisome (91, 92). Important in this review is the D-series resolvins and specifically RvD1. This SPM has overlapping activities with LXA_4_ and acts via the same G-protein-coupled receptors, ALX/FPR2 and GPR32 (Table 1) (63).

## Analytical Approaches to Identify and Measure Lipid Mediators

The identification and quantitation of PUFA-derived lipid mediators have been a challenge due to the small quantities produced within tissues and cells. Thus, highly sensitive methods of gas and liquid chromatography-based separations coupled with detection by mass spectrometry (e.g., GC–MS, GC–MS/MS, LC–MS, and LC–MS/MS) and immunology-based assays [enzyme-linked immunosorbent assay (ELISA)] have played a pivotal role in the analysis of lipid mediators (93, 94).

The separation of individual lipid mediators by GC or LC allows the analyses of multiple lipid mediators in a single biological sample, and the detection of the lipid mediators by MS or MS/MS provides a means for their identification and quantification (95). It is noted that many of the ω3 and ω6 PUFA-derived lipid mediators are isomers, therefore the fragmentation patterns generated my MS/MS provide additional structural information over what is obtained with an accurate mass measurement (MS) (96). However, some isomeric lipid mediators produce similar fragment ion profiles. Thus, it is important to apply authentic standards with rigorous chromatographic separation to confirm the identity of specific lipid mediators. A major advantage of LC–MS or LC–MS/MS as compared to GC–MS or GC–MS/MS is that derivatization to ensure volatility of the lipid mediators is not required (97). Nevertheless, GC-based approaches remain an important tool for confirming the structure and abundance of lipid mediators obtained via LC–MS or LC–MS/MS analyses (93, 94, 98).

Enzyme-linked immunosorbent assay is an orthogonal approach for the quantification of lipid mediators and offers relatively high sensitivity and selectivity (97). However, ELISA-based assays are commercially available for only certain lipid mediators, typically those that are best characterized for their biological activity. Cross-reactivity of antibodies between lipid mediators is a potential limitation of this technique; thus, antibody specificity should be checked with authentic standards (99).

## The Specialized Pro-Resolving RvD1 in Leprosy: Bad with it, Worse without it

### The Potential Role of RvD1 in Down-Modulation of the Immune Response of Leprosy

Amaral et al. revealed that sera levels of RvD1 in BT and LL leprosy patients were similar, but increased in comparison with the sera of healthy individuals (17). Interestingly, after MDT serum levels of RvD1 in BT and LL patients were reduced to those of healthy controls (17). These data indicated that RvD1 is being produced in response to inflammation and possibly also associated with the presence of the pathogen or pathogen products. However, induction of RvD1 production via *M. leprae* infection has not been investigated.

A comprehensive study to define the biological activity of the D-series resolvins (RvD1 and RvD2) and maresin-1 on the adaptive immune response demonstrated that these SPMs reduce the production of IFN-γ and IL-17 by Th1 and Th17 cells, respectively (26). Moreover, RvD1 was shown to promote the *de novo* generation of FoxP3^+^ Treg cells, the expression of CTLA-4 (a surface marker of Treg cells) and IL-10 secretion. The similar levels of RvD1 in BT and LL patients, does not correlate well with this laboratory assessment of RvD1 activity, since BT patients present a strong Th1 and Th17 responses (3, 4, 6) and LL patients are characterized by T-cell anergy and increased frequency of Treg cells (4, 6). Nevertheless, it would be premature to conclude that RvD1 does not participate in the dichotomous immune responses of TT/BT and BL/LL patients. It is possible that the higher level of RvD1 down-modulates the Th1 immune response in TT/BT as well as BL/LL patients. Martins et al. demonstrated that peripheral mononuclear cells (PBMCs) from paucibacillary (TT/BT) leprosy patients possess a lower capacity to produce IFN-γ than healthy individuals exposed to *M. leprae* (3). Thus, the adaptive immune response in TT/BT individuals is still reduced as compared to healthy controls. Furthermore, it could be that RvD1 activity is related to the level of expression of its cognate receptors, GPR32 and ALX/FPR2. Thus, studies that assess the presence of these receptors in the T cells of TT/BT and BL/LL patients are required to fully understand the potential influence of RvD1 on the adaptive immune response across the spectrum of leprosy. Polymorphisms in the promoter region of the ALX/FPR2 gene resulting in a reduced expression of this receptor are known (100, 101). Thus, it would also be interesting to investigate whether polymorphisms exist between TT/BT and BL/LL patients in the promoter or functional regions of the GPR32 and ALX/FPR2 genes.

### RvD1 Regulation of Macrophage Activity: A Possible Factor That Sustains Paucibacillary Infection

**Figure 3:**
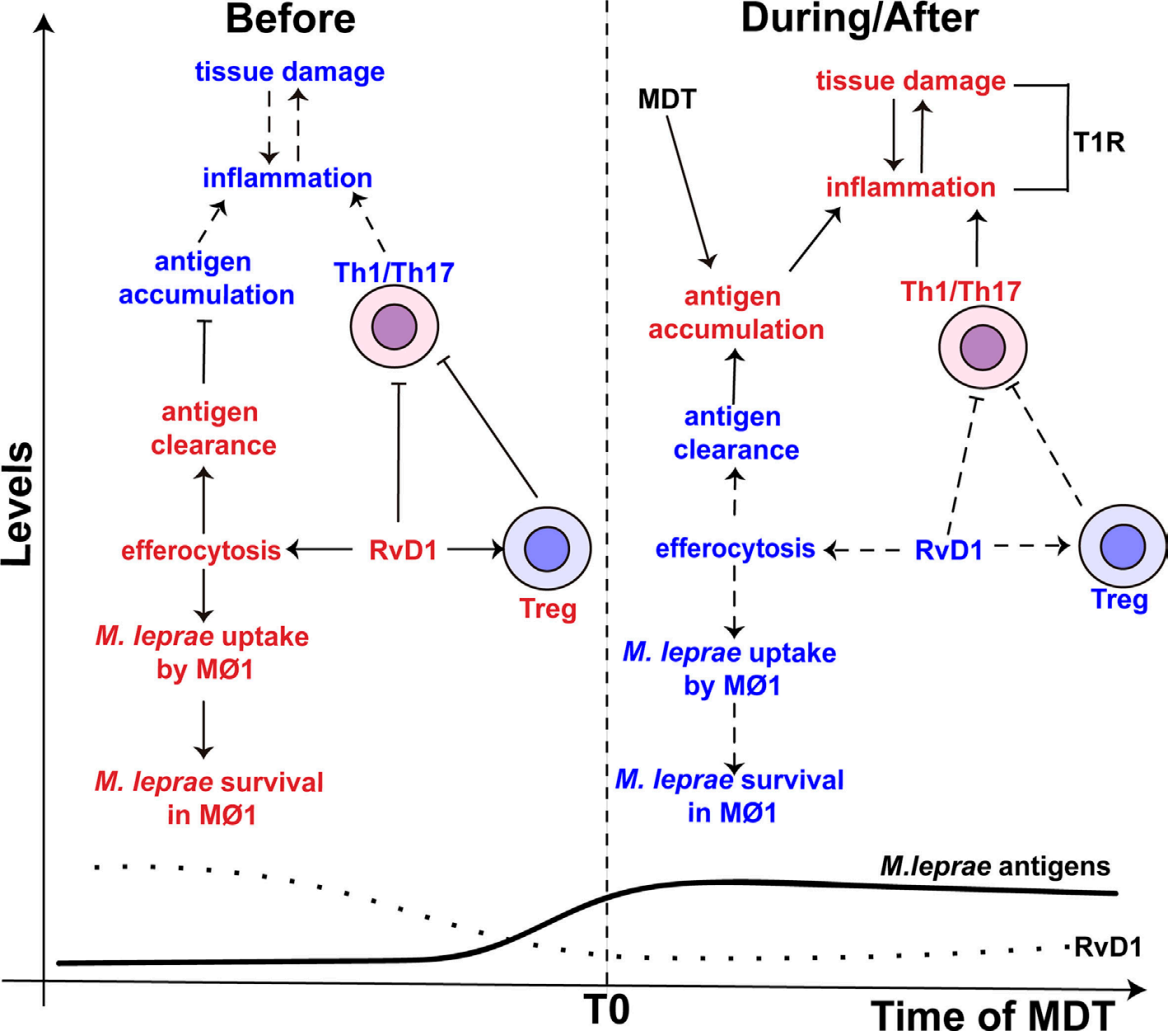
The proposed role of resolvin D1 (RvD1) in leprosy. (Left side) The levels of RvD1 (dotted line) are higher before the start (T0) of multidrug therapy (MDT). The higher levels of RvD1 are hypothesized to increase the host’s susceptibility to *M. leprae* infection. The increased levels of RvD1 prior to MDT could enhance the capacity of macrophages to engulf *M. leprae* antigens as well as the pathogen itself via efferocytosis. This would lead to antigen clearance, decreased antigen stimulation of T-helper type 1 (Th1) and T-helper type 17 (Th17) cells and favor the survival of *M. leprae*. Moreover, increased levels of RvD1 could directly inhibit Th1 and Th17 cells’ response and promote the activity of T regulatory (Treg) cells. (Right side) After the start of MDT, the levels of RvD1 decrease (dotted line), while the abundance of *M. leprae* antigens increase (solid line) due to lysis and degradation of the bacilli, especially in multi-bacillary patients. The reduction of RvD1 could eliminate the suppression of the Th1 and Th17 responses, reduce the activation of Treg cells, and also decrease the ability of macrophages to promote efferocytosis. This impairment in efferocytosis would favor antigen accumulation. Thus, response to mycobacterial antigens by Th1 and Th17 cells would increase resulting in an immune-inflammatory response and potentially a T1R. The red color represents an intensification or increase in a process or abundance of a product, while the blue color symbolizes an attenuation of the process or product abundance. Arrows with solid lines indicate that a process related to the associated RvD1 level is favored, while an arrow with a hashed line indicates the process is not favored. (⊢) Represents inhibition of a process or activity. MΦ1 – M1, pro-inflammatory macrophages.

Besides the ability to reduce the activity of Th1 and Th17 cells, RvD1 also controls the activity of macrophages (102, 103). RvD1 induces efferocytosis in monocytes/macrophages (73), a process that engulfs apoptotic cells and is reported to play an important role in the clearance of *Mycobacterium tuberculosis* and *Mycobacterium avium* (104, 105). However, De Oliveira and colleagues indicated that this process might promote the persistence of *M. leprae* (106). Specifically, in the presence of *M. leprae*, efferocytosis alters the phenotype of the pro-inflammatory M1 macrophage toward anti-inflammatory M2 phenotype with increased the uptake and survival of *M. leprae*. Therefore, in paucibacillary patients, where apoptotic bodies are present in higher number (107, 108), efferocytosis may play an important role in the *in vivo* persistence of *M. leprae*. The increased levels of RvD1 in TT/BT patients could help drive this process (Figure 3).

Adding to the immunomodulatory activity of efferocytosis, it is recognized that *M. leprae* inhibits the capacity of macrophage to respond to IFN-γ stimulation (47) and impairs the production of pro-inflammatory cytokines (e.g., IL-6 and TNF-α) (109). Macrophages infected with *M. leprae* have been found to preferentially prime Treg cells over Th1 or cytotoxic T cells (110). Thus, RvD1 may have an additive or synergistic effect on macrophage function that further reduces the innate responses against *M. leprae* and consequently allows the survival of the pathogen in leprosy patients with a robust Th1 and Th17 cells response (Figure 3). However, studies are required to determine whether RvD1 preferentially drives the response of *M. leprae*-infected macrophage, as well as enhancement of *M. leprae* uptake in the context of efferocytosis. While we would hypothesize that RvD1 would influence macrophage polarization in the context of *M. leprae* infection, the involvement of other lipid mediators in this process cannot be excluded.

### The Reduction of RvD1 Levels in T1R: The Worse

T1R is a major complication in borderline leprosy patients (BT, BB, and BL) and occurs before, during and after MDT (111). The increased inflammation of T1R driven by Th1 and Th17 cells in skin lesions and/or nerves can result in permanent loss of nerve function (112, 113).

A higher bacillary load and MDT are factors associated with the development of T1R pathology (114–116). Thus, it has been hypothesized that the release of *M. leprae* antigens promoted by MDT drive an enhanced immune-inflammatory response, especially in multi-bacillary patients (116, 117). Interestingly, the levels of RvD1 in leprosy patients decrease after the conclusion of MDT (17). Thus, a reduction in circulating SPM may remove suppressive activity being placed on Th1/Th17 cells and contribute to susceptibility of developing T1R in the presence of *M. leprae* antigens (Figure 3). Recently, a metabolomics study of sera from leprosy patients with and without T1R, and that had not started MDT, confirmed that the level of RvD1 was significantly increased (9.01-fold) in non-T1R leprosy patients as compared to T1R leprosy patients and healthy controls (18). These findings indicate a direct correlation with reduced RvD1 levels and destructive inflammation due to enhanced Th1/Th17 activity and revealed that reduced RvD1 production could occur during active disease.

As the balance of pro-inflammatory and pro-resolving lipid mediators are important in the development and control of inflammation, it is important to note that RvD1 also down-regulates the production of the pro-inflammatory lipid mediator LTB_4_ (68). LTB_4_ promotes chemotaxis of Th1 (32) and Th17 cells (33) and enhances the production of pro-inflammatory cytokines associated with Th1 responses (TNF-α and IFN-γ) (22). Although the concentration of LTB_4_ in BT and LL patients are similar to healthy individuals (17), Silva and colleagues observed a significantly increased level of serum LTB_4_ during T1R (18). Studies to define the mechanisms of RvD1 activity revealed that this SPM inhibits the translocation of 5-LO to the nucleus and this inhibits the synthesis of LTB_4_ (68). This mechanism would explain why the levels of LTB_4_ were not increased in leprosy patients without T1R, but with a reduction of RvD1, they become elevated in T1R patients. However, it does not explain why the levels of LTB_4_ did not increase after MDT in leprosy patients without T1R since this treatment reduced RvD1 concentrations (17). It is possible that therapeutic elimination of infection reduces signals and stimuli leading to LTB_4_ production, as well as those that drive RvD1 production.

In conclusion, although increased RvD1 levels may favor *M. leprae* infection by modulating the protective innate and adaptive immune responses (i.e., bad with it), at the same time, RvD1 is likely important to avoid exacerbated inflammation that may cause skin and nerve injuries. Once the levels of the RvD1 drop in a leprosy patient (e.g., because of MDT or other factors), we hypothesize that this increases susceptibility to pathogenic Th1 and Th17 responses against *M. leprae* antigens (i.e., worse without it).

## The Balance Between the Pro-Inflammatory LTB_4_ and the Specialized Pro-Resolving LXA_4_ in Leprosy

### The Higher Levels of LXA_4_ in Leprosy: A Possible Association with the Chronic Nature of *M. leprae* Infection

The study of Amaral et al. demonstrated that LXA_4_ is increased in leprosy patients (17). However, the biological function of LXA_4_ in *M. leprae* infection is not well understood, but has been studied in *M. tuberculosis* infection, another model of chronic infectious disease. In the murine model of tuberculosis, Bafica et al. showed that after 1 week of *M. tuberculosis* infection, LTB_4_ and LXA_4_ increase in abundance as compared to uninfected animals, but the levels of LTB_4_ decrease after 10 days while those of LXA_4_ persist during chronic *M. tuberculosis* infection (20). Interestingly, mice deficient for 5-LO (5-*lo^−/−^*) did not produce LXA_4_ increasing the resistance against *M. tuberculosis* due to higher production of Th1-derived cytokines (INF-γ and IL-12). Conversely, the 5-*lo^−/−^* mice treated with a LXA_4_ analog reduce the levels of Th1 cytokines resulting in increased susceptibility to *M. tuberculosis* (20). These results indicate that LXA_4_ has a more predominant effect than LTB_4_ during *M. tuberculosis* infection and that a high LXA_4_ favors the mycobacterial infection. Similar to the animal studies with *M. tuberculosis*, infection of humans by *M. leprae* and the presentation of leprosy, are associated with increased levels of LXA_4_, but not LTB_4_ (17). This likely reflects the capacity of an *M. leprae* infection to pass unnoticed for years (1–10 years), presumably due to a protective and non-pathogenic immune response. However, as observed for household contacts, a gradual increase in bacillary load and continuous exposure to antigen, down-modulates the immune response against *M. leprae* (3, 118). Thus, we hypothesize that the reduced capacity of the host to respond to *M. leprae*, even during an increase in the bacillary load, is exacerbated by a higher production of LXA_4_. Once this SPM and RvD1 are produced in sufficient amounts they would inhibit the production of LTB_4_ (68), and thus elevated levels of LXA_4_, together with RvD1, might favor the chronic infection of *M. leprae*.

### The Link between LXA_4_/LTB_4_ Ratios and the Expression of TNF-α in Leprosy

It is suggested that LTB_4_ and LXA_4_ modulate the expression or the effects of TNF-α, a pro-inflammatory cytokine involved with the resistance/susceptibility to leprosy (21, 22, 119). Moreover, an imbalance in the ratio of the pro-resolving LXA_4_ to pro-inflammatory LTB_4_ (LXA_4_/LTB_4_) is related with a poor control of the immune-inflammatory response in humans (120, 121). Collectively, metabolomics data produced with sera of leprosy patients indicate that the balance between LXA_4_ and LTB_4_ is altered (17, 18). However, the mechanisms by which altered ratios of LXA_4_/LTB_4_ affect the immunopathology of leprosy remain undefined.

Previous works from Tobin et al. demonstrated that the LXA_4_/LTB_4_ ratio was an important factor in susceptibility of zebrafish larvae to *Mycobacterium marinum*, due to the modulation of TNF-α expression (21, 88, 89). Specifically, shunting LTA_4_ into LXA_4_ synthesis resulted in an increase in the LXA_4_/LTB_4_ ratio and consequently a down-modulation of TNF-α expression (21, 88, 89). This culminated in a high bacterial burden, death of infected macrophages and increase in the severity of the disease. In contrast, accumulation of LTB_4_ enhanced TNF-α expression and enabled macrophage control of infection, but an excess of TNF-α results in the necrosis of macrophages and a higher burden of infection (88, 89). Previous findings support a correlation between the levels of TNF-α and LXA_4_/LTB_4_ ratio in leprosy patients. Both paucibacillary and multi-bacillary leprosy patients exhibited similar levels of TNF-α, LTB_4_ and LXA_4_ (11, 17, 122). On the other hand, leprosy patients with T1R possess a lower LXA_4_/LTB_4_ ratio (18), which agrees with increased inflammation and higher levels of TNF-α observed in these patients (123). Thus, the balance between pro-inflammatory and pro-resolving lipid mediators is important to the outcome of infection.

Furthermore, support for the importance of a LXA_4_/LTB_4_ balance is provided through population genetics in humans (21). Vietnamese and Nepali individuals homozygous for a common promoter polymorphism at the human *LTA4H* locus display lower protection against tuberculosis and multi-bacillary leprosy, respectively. This polymorphism is associated with deficient (low activity alleles) or excessive (high activity alleles) expression of the *LTA4H* gene. Conversely, heterozygous individuals displayed a moderated expression of *LTA4H* gene and consequently a more balanced production of LXA_4_ and LTB_4_, due to the presence of both a low-activity allele and a high-activity allele (21, 88). As a consequence, heterozygous *LTA4H* individuals exhibited better protection against mycobacteria infection.

**Figure 4:**
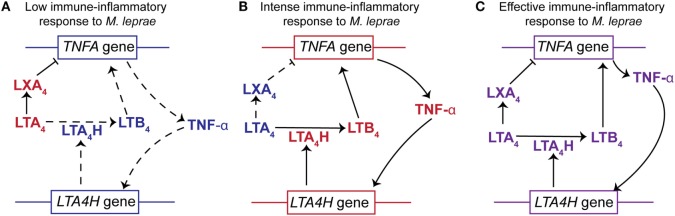
The relationships between *LTA4H* gene polymorphisms, the LXA_4_/LTB_4_ ratios and TNF-α production to the outcome of *Mycobacterium leprae* infection. **(A)** Individuals homozygous for *LTA4H* locus with two low activity alleles display a higher concentration of LXA_4_ than LTB_4_ (high LXA_4_/LTB_4_ ratio). This would impair the production of TNF-α resulting in increased susceptibility to *M. leprae*. The higher levels of LXA_4_ not only inhibit the expression of *TNFA* but also block the immune-inflammatory responses. In addition, the lower levels of TNF-α do not stimulate the expression of *LTA4H* and therefore do not increase the synthesis of LTB_4_. **(B)** Subjects homozygous for *LTA4H* locus with two high activity alleles display a higher concentration of LTB_4_ than LXA_4_ (low LXA_4_/LTB_4_ ratio). The increased abundance of LTB_4_ stimulates the expression of *TNFA* and production of TNF-α. Increased levels of TNF-α further enhance expression of *LTA4H*. Thus, an intense immune-inflammatory response to *M. leprae* would occur resulting in damage to the host tissue. **(C)** Individuals heterozygous for *LTA4H* locus, with a high and a low activity allele, synthesize a balanced amount of LXA_4_ and LTB_4_ (moderated LXA_4_/LTB_4_). This results in the production of TNF-α to levels that promote an effective immune-inflammatory response against *M. leprae* and promote a balance in the LXA_4_/LTB_4_ ratio. This balance in product abundance or gene expression is represented by the purple font. The red font represents an increased abundance of a product or increased gene expression, while the blue font symbolizes an attenuation of product abundance or gene expression. Arrows with solid lines indicate that the production of a lipid mediator or cytokine is favored, while an arrow with a hashed line indicates that the production is not favored. (⊢)Indicates that LXA_4_ attenuates or impairs the expression of TNF-α.

The connection between LTA_4_H and TNF-α is reciprocal, as TNF-α is able to modulate the expression of *LTA4H* (124–126). This suggests that the synthesis of TNF-α and the LXA_4_/LTB_4_ ratio could be regulated by a feedback loop generated by expression of *TNFA* and *LTA4H* (details in Figure 4). Interestingly, polymorphisms in the promoter region of the *TNFA* are associated with human susceptibility to leprosy (119, 127, 128).

Existing data strongly support the hypothesis that the LXA_4_/LTB_4_ ratio in leprosy disease is an important factor in regulation of TNF-α and hence the susceptibility or resistance to *M. leprae* infection. We hypothesize that an increase in the LXA_4_/LTB_4_ ratio leads to lower TNF-α secretion and reduced control of *M. leprae* replication (Figure 4). However, a decrease in LXA_4_/LTB_4_ ratio would promote higher *TNFA* expression and an intense inflammatory response as observed for leprosy patients with T1R.

## A Possible Link Between the Pro/Anti-Inflammatory PGE_2_ and PGD_2_ with Immune Pathological Events in Leprosy Patients

### PGE_2_: A Potential Dual Role in *M. leprae* Infection

**Figure 5:**
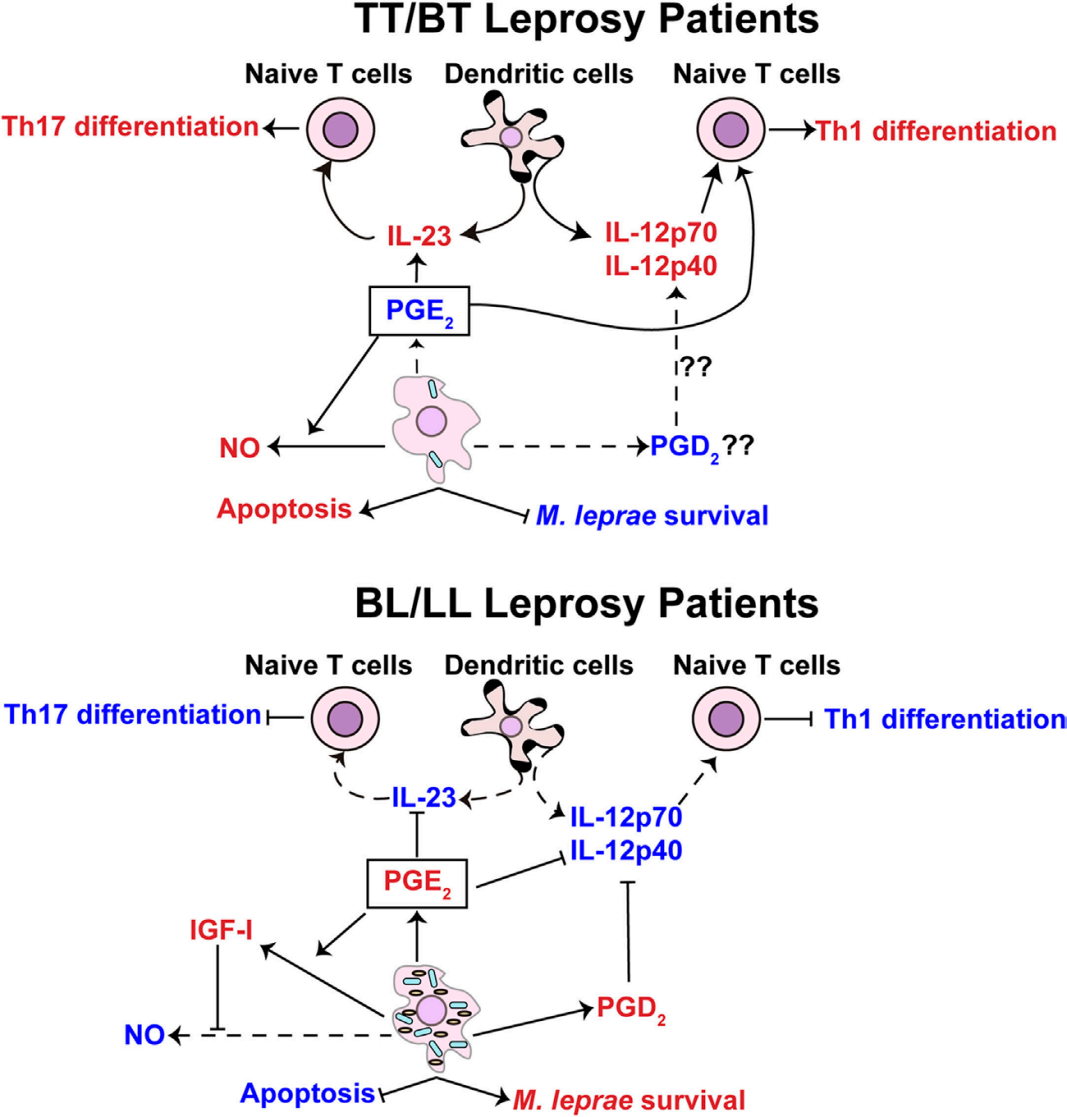
Prostaglandin E_2_ is hypothesized to exhibit different functions in pauci- and multi-bacillary leprosy patients. Tuberculoid (TT)/borderline tuberculoid (BT) leprosy patients (top panel) display a lower concentration of PGE_2_ in comparison with borderline lepromatous (BL)/lepromatous leprosy (LL) patients (lower panel). The lower concentration of PGE_2_ in TT/BT patients is hypothesized to facilitate the differentiation of T-helper type 17 (Th17) cells through upregulation of interleukin (IL)-23 cytokine production by dendritic cells. Findings from Yao et al. (44) provide evidence that small amounts of PGE_2_ may favor the differentiation of T-helper type 1 (Th1) cells in TT/BT individuals. The levels of PGE_2_ in TT/BT patients may also promote the production of nitric oxide (NO) in *M. leprae*-infected macrophages leading to the control of the bacterial load. In BL/LL patients *M. leprae*-infected foamy macrophages/Schwann cells produce a higher level of PGE_2_ that is hypothesized to inhibit the differentiation of Th1 cells through impairment of the production of IL-12p70 by dendritic cells. The higher concentration of PGD_2_, possibly secreted by foamy macrophages/Schwann cells from BL/LL patients, may also inhibit the production of IL-12p70. Additionally, the increased levels of PGE_2_ could potentially inhibit the production of IL-23 in dendritic cells, thus blocking the differentiation of Th17 cells. Increased release of insulin-like growth factor I (IGF-I) stimulated via PGE_2_ might potentially inhibit NO synthesis and apoptosis. The capacity of PGE_2_ to prevent NO production and apoptosis favors the multiplication of *M. leprae*. The red color represents an intensification or increase in a process or abundance of a product, while the blue color symbolizes an attenuation of the process or product abundance. Arrows with solid lines indicate processes (production/secretion of cytokines, helper T-cell differentiation, apoptosis, and/or mycobacteria survival) that are favored or induced, while an arrow with a hashed line indicates processes that are not favored. (⊢) Represents inhibition of a process or activity.

PGE_2_ and PGD_2_ are increased in LL patients (17), and previous studies indicate that foamy macrophages/Schwann cells, a classical hallmark of LL patients, are the main source of prostaglandins (129, 130). The higher levels of PGE_2_ in LL patients (17) together with the lower levels in T1R patients (18) suggest that PGE_2_ is related to the different clinical forms of leprosy. Indeed, this lipid mediator impairs the proliferation of T cells (39, 40) and inhibits the activation of macrophages by IFN-γ in *M. leprae* infection (47). Thus, levels of PGE_2_, produced by foamy macrophages/Schwann cells, can contribute to the inhibition of Th1 responses against *M. leprae* in LL patients. This may also indicate that lower levels of PGE_2_ in T1R patients favors the exacerbated acute responses of Th1 cells. Moreover, PGE_2_ has the ability to augment the suppressive capacity of human CD4^+^CD25^+^ Treg cells and up-regulate the expression of transcription factor *FOXP3* (46). Garg and colleagues demonstrated that PGE_2_, but not PGD_2_, promotes the expansion of Treg cells during *M. tuberculosis* infection (45). Thus, the higher frequency of Treg cells, as well as the anergy of Th1 and Th17 cells in LL individuals, could be related with increased amounts of PGE_2_ secreted by foamy macrophages/Schwann cells (Figure 5). Other mechanisms through which higher levels of PGE_2_ might affect the differentiation of Th17 and Th1 cells in LL patients include, modulating the secretion of IL-23 by dendritic cells (Figure 5) (23) and impairment of IL-12 production by dendritic cells (19).

There is evidence that at the proper concentration and in the presence of a co-stimulatory signal, PGE_2_ also stimulates Th1 response. Yao and colleagues showed that treatment of naive T cells with PGE_2_ and antibody stimulation of CD28 induces the differentiation of Th1 cells (24, 44). It is well known that PGE_2_, through interaction with EP2 and EP4, inhibits the differentiation of Th1 cells by increasing intracellular levels of cAMP (42, 43). However, with a concomitant stimulation of CD28, T cells are rescued from the inhibitory effects of cAMP and therefore differentiate to Th1 cells (24). Interestingly, *M. leprae* antigens are able to reduce the expression of B7-1 and CD28 molecules in PBMC cultures from healthy controls (131), and the levels of B7-1 and CD28 molecules in BL/LL patients, but not in BT patients, are reduced. Therefore, the higher levels of PGE_2_ that leads to an increase in the intracellular levels of cAMP together with lower expression of CD28 could inhibit the differentiation of Th1 cells in LL patients. Conversely, BT patients that secrete basal levels of PGE_2_ and express higher levels of CD28 would be expected to propagate and maintain a Th1 response. T1R patients also exhibit a basal level of PGE_2_ (18). Hence, our hypothesis is that lower PGE_2_ levels promote Th1 and Th17 cell activities in BT and T1R patients, but in LL patients, the higher concentration of this prostaglandin inhibits Th1 and Th17 responses (Figure 5). Together, these studies highlight the controversial role of PGE_2_ in the human adaptive immune response and underscore the need for studies to determine other possible roles of PGE_2_ in leprosy.

### The Control of NO Production by PGE_2_

The prostaglandin PGE_2_ has been shown to also interfere with the control of cell death (48) and the production of NO by phagocytic cells (41). Studies using an experimental animal model of pulmonary tuberculosis demonstrated that at the early phase of *M. tuberculosis* infection, BALB/c mice produce lower amounts of PGE_2_ and this promotes the expression of the inducible form of NO synthase (*iNOS*). In contrast, at later stage of infection, higher amounts of PGE_2_ are produced and inhibit the expression of *iNOS* (41). These assays support the idea that lower production of PGE_2_ favors the bacterial control, and at higher concentrations, PGE_2_ inhibits microbicidal mechanisms in the murine model. In line with these observations, skin lesions of BT leprosy patients exhibit a higher expression of *iNOS* than those of BL patients (11), and macrophages isolated from BT patients secrete higher concentrations of nitrite, a marker for iNOS activity, than macrophages derived from LL patients (132). Thus, we hypothesize that the lower levels of PGE_2_ in BT patients (17) directly promote the microbicidal activities of phagocytic cells to control *M. leprae* replication as well as enhance the Th1 responses. Interestingly, the higher production of NO may cause nerve damage in BT patients as hypothesized in previous work (15). On the other hand, higher concentrations of PGE_2_ secreted by foamy macrophages/Schwann cells would inhibit these same antimicrobial activities and thus favor multi-bacillary disease (Figure 5).

### PGE_2_ Might Differently Influence Apoptosis in Tuberculosis and Leprosy Patients

A potential mechanism by which PGE_2_ would inhibit the production of NO in LL patients is through the induction of insulin-like growth factor I (IGF-I). PGE_2_ induces the expression of IGF-I in murine macrophages (133) and osteoblasts (134, 135), and IGF-I inhibits the NOS2 pathway (136). A recent study has demonstrated that increased amounts of IGF-I are found in the skin lesions of LL patients and that IGF-I inhibits signaling cascades required for NO production (137). Therefore, it is possible that the elevated levels of PGE_2_ could be linked to the inhibition of NO production via the induction of IGF-I in LL patients.

The production of IGF-I, possibly mediated by PGE_2_, may also promote *M. leprae* survival by inhibition of apoptosis. Live *M*. *leprae* induces the production of IGF-I in Schwann cells and this was found to prevent apoptosis (138). The inhibition of apoptosis could be a significant advantage for *M. leprae* since this mechanism of cell death promotes the presentation of mycobacterial antigens to T cells (139). Thus, via an IGF-I network, PGE_2_ may directly impact antigen presentation and favor *M. leprae* replication (Figure 5). However, a direct functional link between increased IGF-I and PGE_2_ levels in LL individuals and apoptotic activity needs to be experimentally established.

It is interesting to highlight that the role of PGE_2_ in *M. leprae* infection may greatly differ from the function of PGE_2_ during *M. tuberculosis* infection. It appears that, during the early phase of infection, virulent *M. tuberculosis* (H37Rv) inhibits the synthesis of PGE_2_, by inducing synthesis of LXA_4_, to prevent apoptosis and consequently inhibit early T-cell activation and promote necrosis of macrophages (48, 49, 139, 140). In contrast, at the chronic stage, PGE_2_ is highly produced (41), which could control the bacillary load by apoptosis. Furthermore, macrophages infected by the avirulent strain of *M. tuberculosis* (H37Ra) produced increased levels of PGE_2_ (48), promoting the protection against mitochondrial inner membrane perturbation and induced plasma membrane repair, crucial processes to avoid necrosis and induce apoptosis (48, 49). Thus, PGE_2_ might be crucial for the resistance against *M. tuberculosis* but promote susceptibility to *M. leprae*. These possible differences between *M. tuberculosis* and *M. leprae* infections could be partially related with different modulation of EP1-4 receptors by the two pathogens and should be explored in future studies.

### PGD_2_ in Leprosy: A Lipid Mediator Exploited by the Pathogen or a Host Response to Nerve Damage

Based on the several findings regarding PGD_2_ and its effects on the modulation of T cells we suggest that PGD_2_ production via foamy macrophages/Schwann cells promotes Th2 response in LL patients. It is well established that PGD_2_ decreases the numbers of CD4^+^ and CD8^+^ T cells that produce IFN-γ and IL-2, through interactions with the DP1 receptor, while contributing to the Th2 responses with induction of IL-4, IL-5, and IL-13 by binding the CRTH2 receptor (60, 61). Besides a direct effect on T cells, PGD_2_ modulates the T-cell response through dendritic cells and their production of IL-12 (19, 59). Braga et al. has revealed that monocyte-derived dendritic cells from LL patients produced less IL-12 (25), and although a direct association has not been made, the decreased IL-12 levels in LL patients could be driven by increased PGD_2_ production and secretion by foamy macrophages/Schwann cells (Figure 5).

One observation that does not fit with the PGD_2_ immune suppressing scenario in leprosy is that PGD_2_ levels increase during a T1R (18). T1R is considered a delayed type hypersensitivity (DTH) reaction (141) and several works indicate that PGD_2_, or its metabolite 15d-PGJ_2_ (142), is highly produced during DTH to control the inflammatory activity in animal models (143). Thus, the increasing of PGD_2_ in T1R patients may be a response by the host to control inflammation.

Individuals with acute inflammatory demyelinating polyneuropathy, an autoimmune disease that directly attack the peripheral nerve myelin (144), have increased levels of PGD synthase enzyme in their cerebrospinal fluid (145). In a murine model of spinal cord contusion injury, the levels of PGD synthase are also elevated (146). Interestingly, although the expression of PGD synthase was never determined, COX-2 is increased during T1R (147, 148). Thus, an increase in PGD_2_ is not unexpected during T1R as these leprosy patients suffer the most severe nerve damage. PGD_2_ is known to promote the myelination of neurons (55). In addition, mice that lack PGD synthase are unable to promote myelination of the neurons. These studies, as well as the fact that mast cells that are in close proximity to the peripheral nerve fibers in the tissue are the major producers of PGD_2_, support the hypothesis that increased PGD_2_ is a consequence of the T1R in leprosy and not a driver of the pathology.

Given the potentially varied activities of PGD_2_ at different stages of leprosy, it is important to determine not only the source of this prostaglandin, foamy macrophages/Schwann cells versus mast cells, but also the receptors that bind PGD_2_ during the different manifestations of leprosy and the cells that are expressing these receptors. Additionally, PGD_2_ potentiates the formation of edema (56, 57), a factor that might contribute to the nerve damage in leprosy (149). Therefore, further studies are required to determine if PGD_2_, through edema formation, can contribute to the pathology of leprosy lesions.

## Summation and Conclusion

Through the multiple metabolomics studies performed with clinical samples from leprosy patients it is clear that alterations in the metabolism of lipid mediators derived from ω3 and ω6 PUFA occur with this disease. However, there is a lack of research that directly links these lipid mediators to the breadth of immune responses that occur across the clinical manifestations of leprosy. Detailed investigations to define enzymes and biochemical pathways for lipid mediator synthesis, along with elucidation of lipid mediator receptors and mechanisms by which lipid mediators influence both innate and adaptive immune responses, has nevertheless allowed the development of well supported hypothesis on the function of various lipid mediators in different manifestations of leprosy. A common theme that has emerged from existing studies is that several of the lipid mediators identified in the metabolomics studies of leprosy patients and discussed here (RvD1, LXA_4_, PGE_2_, and PGD_2_) down-regulate the immune-inflammatory responses promoted by Th1 and Th17 cells and facilitate the activity and proliferation Treg cells. This would indicate that *M. leprae* might exploit the pro-resolving activities of lipid meditators to maintain a persistent infection. Nonetheless, some of these lipid mediators such as PGE_2_ and PGD_2_, as well as LTB_4_ can influence the protective response against *M. leprae*. Another emerging theme is that alteration of the balance between pro-inflammatory and pro-resolving lipid mediators has the potential to dramatically skew the Th1/Th17 and Treg responses in leprosy. This same concept also applies to variations in the relative concentration of individual products such as PGE_2_. Thus, a coordination of the dynamics of the lipid mediator response and that of the adaptive and innate immune systems seems to be a driving factor in the specific presentation of leprosy.

As existing and future data are interpreted to develop models of lipid mediator involvement in the pathology and immunology of leprosy, it is important to consider the complexity of lipid mediator metabolism, and that most lipid mediators can serve as ligands for multiple receptors. Additionally, the spatial and temporal aspects of lipid mediator metabolism and receptor expression, along with the complementary or opposing activities of multiple lipid mediators must be addressed to fully elucidate the role lipid mediators play in leprosy. Mathematical models, as performed for *M. tuberculosis* infection (150), may be important to elucidate the influence PUFA-derived lipid mediator complexity in disease outcomes that might occur in individuals infected with *M. leprae*. It is also important to highlight that lipid mediators not identified or targeted in previous metabolomics studies on leprosy, may also contribute to immuno-pathogenesis. Thus, further targeted metabolomics investigations supported by orthogonal approaches, such as transcriptomics and proteomics, are needed to elucidate the full complement lipid mediators involved in leprosy and define how systemic alterations in their levels modify the phenotype of innate and adaptive immune cells in different presentations of leprosy. Future research efforts will not only provide an understanding of the contribution of lipid mediators to chronic infectious diseases but also provide the basis for the development of new diagnostic/prognostic and treatment approaches to address leprosy as a public health problem.

## Author Contributions

CS and JB contributed to the review of published literature, development of the concepts, and design of the review article, as well as the writing and editing of the manuscript. CS is responsible for the design and concepts of the figures.

## Conflict of Interest Statement

The authors declare that the research was conducted in the absence of any commercial or financial relationships that could be construed as a potential conflict of interest. The reviewer OM and handling editor declared their shared affiliation.
